# Identifying Travel Regions Using Location-Based Social Network Check-in Data

**DOI:** 10.3389/fdata.2019.00012

**Published:** 2019-06-12

**Authors:** Avradip Sen, Linus W. Dietz

**Affiliations:** Department of Informatics, Technical University of Munich, Munich, Germany

**Keywords:** data-mining, human mobility modeling, spatial clustering, region detection, visualization

## Abstract

Travel regions are not necessarily defined by political or administrative boundaries. For example, in the Schengen region of Europe, tourists can travel freely across borders irrespective of national borders. Identifying transboundary travel regions is an interesting problem which we aim to solve using mobility analysis of Twitter users. Our proposed solution comprises collecting geotagged tweets, combining them into trajectories and, thus, mining thousands of trips undertaken by twitter users. After aggregating these trips into a mobility graph, we apply a community detection algorithm to find coherent regions throughout the world. The discovered regions provide insights into international travel and can reveal both domestic and transnational travel regions.

## 1. Introduction

The destinations visited within a trip may overarch existing administrative divisions of provinces, federal states, and countries. For example, visiting the Alps of Europe, one is not restricted in travel by country borders as all adjacent countries are members of the Schengen Area. When developing a travel region recommender system for composite trips this is a challenge, because one needs a region model to choose the recommendations from Dietz (2018). To come up with such a model, we propose to observe traveler mobility behavior, aggregate it using spatial clustering methods, thereby re-drawing the boundaries of the world's travel regions using a data-driven approach.

Data collected from location-based social networks has previously been used as a proxy for human mobility, however, such data sets are either not readily available, are focused on small areas, such as cities, or have too sparse check-ins of the users. Hence, we use public Twitter APIs to collect traveler data in the form of geotagged tweets. From the series of tweets, we determine the home location of the user and then extract the trips (Dietz et al., 2018). These trips are then aggregated into a weighted graph of tourist flows with nodes being cities and edges being the number of trips from one city to another. This graph is then fed into a community detection algorithm (Bohlin et al., 2014), whose results constitute the world's travel regions irrespective of established political boundaries.

In this position paper, we want to motivate this approach, describe our ideas to implement and evaluate such a method. Furthermore, we outline the implications and benefits of a data-driven region model in other domains, such as recommender systems.

## 2. Method

Twitter allows algorithmic access to a stream of public tweets through their APIs, which can be queried to build a data set of geotagged tweets. By querying timelines of users who have enabled sharing the geolocation of their tweets, we can follow their movement patterns. To reduce noise, the individual geolocations are matched to the nearest city. Thus, each tweet in the timeline constitutes a check-in to a city. After the home city of the user has been determined by the highest number of check-ins, consecutive check-ins outside of the home city can then be combined to a trip. To focus on travelers, we exclude all trips shorter than 7 days. Furthermore, we require at least one check-in within 5 days, to ensure sufficient data quality. For more details on the trip mining, we refer to our previous paper (Dietz et al., 2018).

The trips are then transformed into an undirected graph, where each city is a node, and the edges represent the flows divided by the distance between the two cities. The flows are computed by summing up the co-occurrences of the two nodes in a clique formed by all cities in a trip. For example, if somebody traveled from Munich to Berlin via Nuremberg in one trip, we would also count the flow from Munich to Berlin as one. Including the distance into the edge weight was useful to reduce noise in the flow graph introduced by distant traffic hubs, such as airports. With this graph-based representation, we can run the Infomap multi-level community detection algorithm to see which cities form coherent clusters (Rosvall et al., 2009).

## 3. Preliminary Results

Running this approach with trips from Twitter reveals four major clusters on the highest hierarchy:

1. North and Central America,
2. South America,
3. Europe, Russia, Arabia, Western and South Africa, and
4. Eastern Africa, Asia, and Oceania.

**Figure 1:**
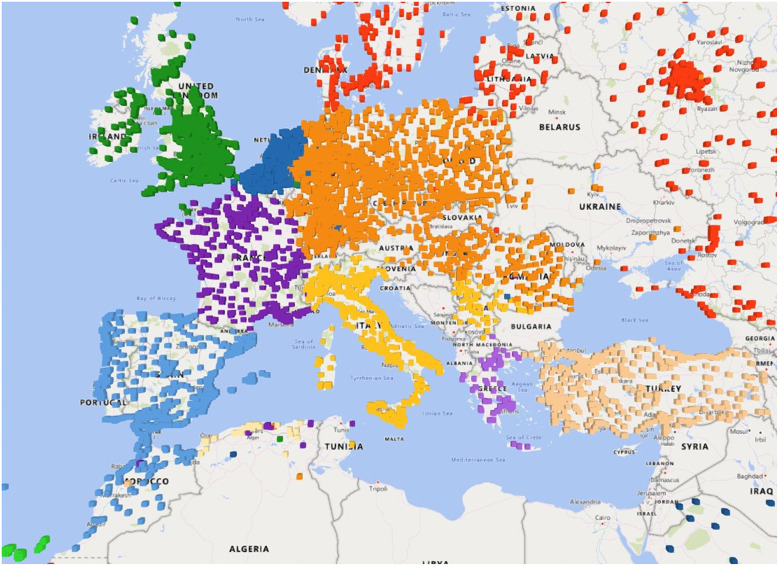
The second-level community structure of Europe.

The level two clusters of Europe, depicted in Figure 1, correspond to groups of similar countries. The British Isles, the Iberian Peninsula, and much of Central and Eastern Europe are merged into respective clusters, while countries like France, Italy, and Turkey roughly retain their own clusters. This is already an interesting result, as it shows that political boundaries have a strong influence on the travel behavior. Subdividing these clusters reveals further regions, however the results become more fuzzy and subject to thorough evaluation. One major challenge is to find a termination criterion to decide whether to continue splitting these clusters. In our opinion, this cannot be decided with the current data, but requires further analysis of the regions, such as the number of cities and the area covered. An evaluation of the quality of the discovered region will also prove to be challenging. However, comparing our third-level clusters of the United Kingdom with those of Ratti et al. (2010) revealed high similarities.

## 4. Related Work

Human mobility analysis has helped us to improve our understanding of traffic forecasting (Kitamura et al., 2000), the spread of diseases (Eubank et al., 2004), and also computer viruses (Kleinberg, 2007). Researchers have already attempted to define regions based on human mobility data for various purposes such as administrative region discovery (del Prado and Alatrista-Salas, 2016), topical region discovery (Taniguchi et al., 2015), and political redistricting (Joshi et al., 2009). Closest to our approach is the work of Hawelka et al. (2014), who aim to find larger regions of mobility, by combining several countries. We aim to find touristic regions that are smaller and potentially independent of countries.

There are various algorithms to perform spatial clustering and community detection, such as the Louvain method (Blondel et al., 2008), GDBSCAN (Ester et al., 1996), and Infomap (Rosvall et al., 2009). They are comparable in runtime complexity, however (Fortunato and Hric, 2016) finds that the Infomap algorithm outperforms the Louvain method in the quality of the communities. GDBSCAN uses the distance between points explicitly to form clusters that are geographically contiguous. Thus, we use Infomap, as it allows to use self-computed weights for the graph and can detect hierarchies. This resolves the resolution limit problem, where the size of communities depend on the size of the graph, which can result in recognized communities being merged together in large networks.

## 5. Conclusions

This position paper introduces an approach for spatial clustering of touristic regions from trips mined from Twitter. To the best of our knowledge, this is the first application of geo-located tweets to find travel regions, with data spanning the whole world. The analysis of results finds a coherent hierarchy of clusters. This confirms that the use of tweets to find traveler mobility patterns and define regions based on the patterns is a feasible approach.

In future, we plan to make a thorough evaluation of the resulting regions using numeric method, but also to visually compare them to findings of other region discovery approaches.

## Data Availability

The raw data supporting the conclusions of this manuscript will be made available by the authors, without undue reservation, to any qualified researcher.

## Author Contributions

AS: Prototype implementation, experimentation, literature analysis. LD: Main author of manuscript, developed the trip mining library.

### Conflict of Interest Statement

The authors declare that the research was conducted in the absence of any commercial or financial relationships that could be construed as a potential conflict of interest. The handling editor and reviewer SG declared their involvement as co-editors in the Research Topic, and confirm the absence of any other collaboration.
